# MyD88-dependent pro-interleukin-1β induction in dendritic cells exposed to food-grade synthetic amorphous silica

**DOI:** 10.1186/s12989-017-0202-8

**Published:** 2017-06-23

**Authors:** Hans Christian Winkler, Julian Kornprobst, Peter Wick, Lea Maria von Moos, Ioannis Trantakis, Elisabeth Maria Schraner, Barbara Bathke, Hubertus Hochrein, Mark Suter, Hanspeter Naegeli

**Affiliations:** 1 Institute of Pharmacology and Toxicology, University of Zurich-Vetsuisse, Winterthurerstrasse 260, 8057 Zurich, Switzerland; 2 Laboratory for Particles-Biology Interactions, Empa Swiss Laboratories for Materials and Technology, Lerchenfeldstrasse 5, 9014 St. Gallen, Switzerland; 3 Department of Health Sciences and Technology, ETH Zurich, Schmelzbergstrasse 9, 8092 Zurich, Switzerland; 4 Electron Microscopy, Institutes of Veterinary Anatomy and Virology, Winterthurerstrasse 260, 8057 Zurich, Switzerland; 5 Department of Research, Bavarian Nordic GmbH, 82152 Martinsried, Germany; 6 Immunology Division, Vetsuisse Faculty, University of Zurich, Winterthurerstrasse 204, 8057 Zürich, Switzerland; 7 Present address: Institute of Food, Nutrition and Health, Laboratory of Human Nutrition, ETH Zurich, Schmelzbergstrasse 7, 8092 Zurich, Switzerland

**Keywords:** E 551, Food additive, Food toxicology, Gut-associated lymphoid tissue, Inflammatory bowel disease, Nanomaterial, Silicon dioxide, Synthetic amorphous silica

## Abstract

**Background:**

Dendritic cells (DCs) are specialized first-line sensors of foreign materials invading the organism. These sentinel cells rely on pattern recognition receptors such as Nod-like or Toll-like receptors (TLRs) to launch immune reactions against pathogens, but also to mediate tolerance to self-antigens and, in the intestinal milieu, to nutrients and commensals. Since inappropriate DC activation contributes to inflammatory diseases and immunopathologies, a key question in the evaluation of orally ingested nanomaterials is whether their contact with DCs in the intestinal mucosa disrupts this delicate homeostatic balance between pathogen defense and tolerance. Here, we generated steady-state DCs by incubating hematopoietic progenitors with feline McDonough sarcoma-like tyrosine kinase 3 ligand (Flt3L) and used the resulting immature DCs to test potential biological responses against food-grade synthetic amorphous silica (SAS) representing a common nanomaterial generally thought to be safe.

**Results:**

Interaction of immature and unprimed DCs with food-grade SAS particles and their internalization by endocytic uptake fails to elicit cytotoxicity and the release of interleukin (IL)-1α or tumor necrosis factor-α, which were identified as master regulators of acute inflammation in lung-related studies. However, the display of maturation markers on the cell surface shows that SAS particles activate completely immature DCs. Also, the endocytic uptake of SAS particles into these steady-state DCs leads to induction of the pro-IL-1β precursor, subsequently cleaved by the inflammasome to secrete mature IL-1β. In contrast, neither pro-IL-1β induction nor mature IL-1β secretion occurs upon internalization of TiO_2_ or FePO_4_ nanoparticles. The pro-IL-1β induction is suppressed by pharmacologic inhibitors of endosomal TLR activation or by genetic ablation of MyD88, a downstream adapter of TLR pathways, indicating that endosomal pattern recognition is responsible for the observed cytokine response to food-grade SAS particles.

**Conclusions:**

Our results unexpectedly show that food-grade SAS particles are able to directly initiate the endosomal MyD88-dependent pathogen pattern recognition and signaling pathway in steady-state DCs. The ensuing activation of immature DCs with de novo induction of pro-IL-1β implies that the currently massive use of SAS particles as food additive should be reconsidered.

**Electronic supplementary material:**

The online version of this article (doi:10.1186/s12989-017-0202-8) contains supplementary material, which is available to authorized users.

## Background

Dendritic cells (DCs) develop from hematopoietic progenitors of the bone marrow under the direction of feline McDonough sarcoma-like tyrosine kinase 3 ligand (Flt3L, Additional file 1: Figure S1). This growth factor is required to generate steady-state DCs that, under homeostatic conditions, reside in lymphoid organs like spleen and migrate to non-lymphoid organs including skin, lung and intestines [1, 2]. Steady-state DCs are dispersed throughout the intestinal mucosa where, in their dual antigen-presenting role, they initiate immune reactions in response to pathogenic agents while maintaining tolerance to self-antigens, innocuous food constituents and the beneficial microbiome [3, 4]. To detect foreign structures and distinguish them from self-antigens, DCs are equipped with a plethora of innate sensors such as Toll-like receptors (TLRs) and Nod-like receptors (NLRs). These pattern recognition receptors are strategically positioned to simultaneously monitor the cell surface, endosomes and cytoplasm [5]. For almost all TLRs (except TLR3), an adapter protein known as myeloid differentiation primary response gene 88 (MyD88) mediates activation of transcription factor NF-κB, which in turn leads to upregulation of cell surface maturation markers and induction of pro-inflammatory cytokines [6, 7].

Although the respiratory toxicity of inhaled nanomaterials gained much attention [8, 9], the gastrointestinal tract is exposed to comparably larger amounts of inorganic particles including nanostructured food additives [10–13]. Silica has currently the highest production volume of all engineered nanomaterials worldwide [14]. Common applications in the food industry use silica in the amorphous rather than crystalline form. In particular, synthetic amorphous silica (SAS) is widely employed as anticaking agent in powdered products, as defoaming agent in beverages, as a thickener in pastes or carrier of flavorings [15, 16]. TiO_2_, containing a minor proportion of particles with a size <100 nm, serves as whitening agent in food and toothpastes [17]. New emerging uses in food include FePO_4_ particles for iron fortification [18, 19]. Both crystalline and amorphous silica lead to acute adverse reactions of the lung after inhalation involving the cytokine interleukin (IL)-1β [9, 20, 21], and synergism between silica and TiO_2_ particles has been reported to exacerbate toxicity in lung macrophages [22]. Because of its potent pro-inflammatory action, IL-1β biogenesis is tightly regulated. First, an inactive pro-IL-1β precursor is synthesized, which is subsequently cleaved by the inflammasome complex (comprising caspase 1 and NLRP3) to yield biologically active IL-1β for extracellular secretion [23–25]. The actual regulator of pro-IL-1β induction remains unclear, but in acute lung inflammation a case has been made for IL-1α, released mainly from necrotic macrophages, being a trigger of pro-IL-1β expression [26]. Another previous report implicated tumor necrosis factor-α (TNF-α) as a pro-IL-1β inducer [27].

SAS particles withstand gastrointestinal digestion [16], reach the intestinal mucosa, penetrate through the mucus and epithelial barriers and accumulate in underlying tissues [28]. Consequently, particle aggregates containing silicon are detected in the gut-associated lymphoid tissue of humans [29, 30]. In addition, silica levels up to 300 μg g^−1^ tissue (a value converted from the measured silicon concentration), were found in the spleen of rodents after repeated oral administration of SAS particles [31]. The intestine is less sensitive to irritation compared with lung, but it has been proposed that a life-long contact of the gut-associated lymphoid tissue with deposits of exogenous particles may lead to harmful long-term reactions responsible for chronic inflammatory diseases of the intestinal tract [11, 28]. To address this potential hazard, we tested the response of steady-state DCs, representing exquisitely sensitive sentinels of foreign materials, to two kinds of food-grade SAS particles currently available on the market. As comparators, we assessed TiO_2_ and FePO_4_ nanoparticles of different sizes. This study was instigated by the notion that steady-state DCs, in view of their specialized function and repertoire of pattern recognition receptors, may respond to nanomaterials by different mechanisms than macrophages or other previously tested cell types.

## Results

### Characterization of particles

High-production examples of commercially available SAS particles intended for the use in food are Aerosil 380F and Aerosil 200F [15, 16, 31]. These food-grade SAS particles were analyzed in depth to determine their shape, specific surface area and hydrodynamic diameter (Table 1 and Additional file 1: Figure S2). Under the conditions used for the testing of cellular responses, i.e., in cell culture medium, the two nanostructured SAS materials display primary particle sizes of 7 and 13 nm, but form aggregates with very similar mean diameters of 147 and 127 nm. Nanoparticles of TiO_2_ or FePO_4_ were included in order to have at hand a range of defined probes for the comparison of DC reactivity towards different nanomaterials. These reference particles of TiO_2_ or FePO_4_ form aggregates ranging in size between 67 and 352 nm. The dissolution of FePO_4_ nanoparticles in aqueous solutions is very low (< 5%) at pH ≥ 2 [19]. Traceable 100-nm polystyrene (PS) particles were used exclusively as a size standard for hydrodynamic diameter measurements. Based on a sensitive *Limulus* amoebocyte lysate assay, all nanomaterials listed in Table 1 were free of endotoxin contamination except a batch of commercial 50-nm PS particles not used for subsequent biological assays.

**Table 1:** Particle characterization

Particle description^*a*^	Specific surface area^*b*^ [m^2^ g^−1^]	Hydrodynamic diameter [nm] in H2O^*c*^ in CM^*d*^		Shape^*e*^	Endotoxin contamination^*f*^ [EU per 250 μg particles]
7-nm SAS	326	147 (105–193)^*g*^	147 ± 5	Irregular	< LOD^*h*^
13-nm SAS	175	182 (130–241)	127 ± 1	Irregular	< LOD
11-nm FePO_4_	188	183 (124–267)	255 ± 35	Irregular	< LOD
21-nm FePO_4_	98	178 (110–258)	230 ± 40	Irregular	0.006
33-nm TiO_2_	47	205 (91–335)	67 ± 18	Almost spherical	< LOD
140-nm TiO_2_	11	Not measured	352 ± 6	Almost spherical	< LOD
50-nm PS-NH_2_^*i*^	Not measured	Not measured	Not measured	Spherical	0.043
100-nm PS^*k*^	Not measured	104(91–114)	Not measured	Spherical	Not measured

^*a*^The primary particle diameter was calculated from specific surface area and weight (2.6 kg m^−3^ for SAS, 2.9 kg m^−3^ for FePO_4_ and 3.9 kg m^−3^ for TiO_2_)

^*b*^Calculated from nitrogen adsorption (Micromeritics Tristar 3000) at 77 K and relative pressure range p/p0 = 0.05–0.25 using the Brunauer-Emmett-Teller (BET) theory

^*c*^Determined using Nanoparticle Tracking Analysis 2.3 on a NanoSight instrument (Malvern)

^*d*^Determined by dynamic light scattering using a Zetasizer Nano ZS (Malvern). CM, complete cell culture medium; values are reported as mean ± standard deviation (*n* = 3) and remained stable during 24 h

^*e*^Determined by transmission electron microscopy (TEM)

^*f*^Determined in the Endosafe PTS endotoxin test (Charles River). A control with *Escherichia coli* LPS at the concentration of 10 pg ml^−1^ yielded 0.037 EU ml^−1^; EU, endotoxin units

^*g*^Numbers in parenthesis show the 10% and 90% probability range

^*h*^LOD, limit of detection (0.005 EU ml^−1^)

^*i*^This endotoxin finding obtained with a purchased batch of polystyrene (PS) particles is included to show an example of contaminated commercial material

^*k*^These 100-nm traceable standard particles from Thermo Scientific served as a size standard for hydrodynamic diameter measurements by Nanoparticle Tracking Analysis

### Steady-state DCs internalize food-grade nanomaterials

**Fig. 1:**
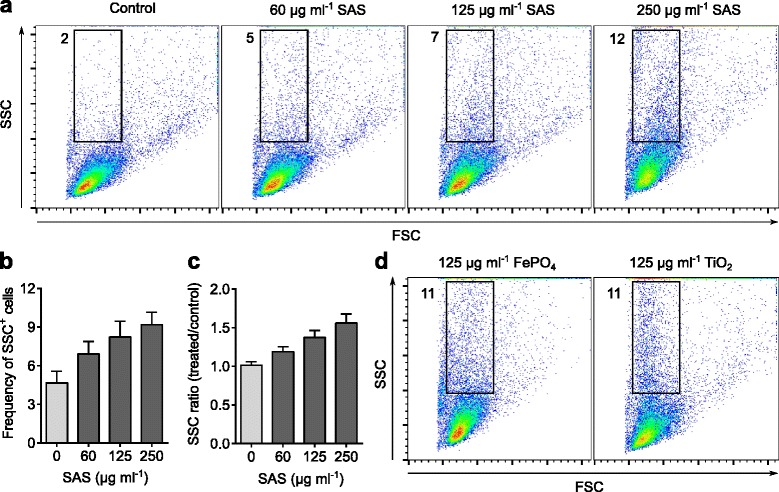
Interaction of steady-state DCs with nanomaterials. Flt3L-generated immature DCs were incubated for 1 h at 37 °C with the indicated concentrations of SAS (13-nm primary diameter), 11-nm FePO_4_ or 33-nm TiO_2_ particles, and analyzed by flow cytometry. In culture medium, the SAS particles form aggregates with a mean diameter of 127 nm. The forward scatter (FSC) depends on cell size whereas the side scatter (SSC) reflects intracellular contents like granules [34, 35]. **a** Flow cytometry distributions demonstrating a SAS dose-dependent increase of DCs with elevated SSC values (numbers denote percentages of events in each gate). **b** Mean percentage of cells in the selected gate (shown in **a**) with high SSC values. Upon one-way ANOVA, SAS treatments increased the proportion of high-SSC cells in a significant manner (*p* < 0.05, *n* = 4 experiments with independent bone marrow isolates). Error bars, standard errors of the mean (s.e.m.). **c** Ratios of median SSC. Upon one-way ANOVA, SSC values after incubation with SAS particles were significantly higher than controls (*p* < 0.05, *n* = 4). **d** Comparison with SSC increments resulting from incubation of DCs with FePO_4_ and TiO_2_ nanoparticles (quantifications are shown in Additional file 1: Figure S3)

Mouse bone marrow cells were incubated with Flt3L to generate immature DCs (Additional file 1: Figure S1). The in vivo correlates of these Flt3L-generated populations are lymphoid tissue-resident DCs found under steady-state conditions [2, 32, 33]. These immature DCs were maintained in culture flasks or wells as cell suspensions and their interaction with SAS particles (displaying a primary particle size of 13 nm), FePO_4_ particles (with a primary size of 11 nm) and TiO_2_ particles (with a primary size of 33 nm), also suspended in cell culture medium, was monitored by flow cytometry. The resulting side scatter (SSC) has been reported to reflect internal cellular structures due to particle uptake whereas the front scatter (FSC) represents cell size [34, 35]. Upon incubation with SAS particles, the proportion of DCs with elevated SSC values indicative of particle uptake was increased in a dose-dependent manner (Figs. 1a-c). In contrast, the front scatter (FSC) and, hence, cell dimensions remained unchanged when the DCs were exposed to SAS particles. Similar responses with elevated SSC and unchanged FSC were observed upon incubation of DCs with FePO_4_ and TiO_2_ nanoparticles (Fig. 1d, Additional file 1: Figure S3). These findings indicate that immature DCs readily interact with all three types of nanomaterials resulting in particle internalization. Fig. 1a and d show that a lower concentration (125 μg ml^−1^) of FePO_4_ and TiO_2_ nanoparticles is needed to achieve the same SSC shift as with 250 μg ml^−1^ SAS particles.

**Fig. 2:**
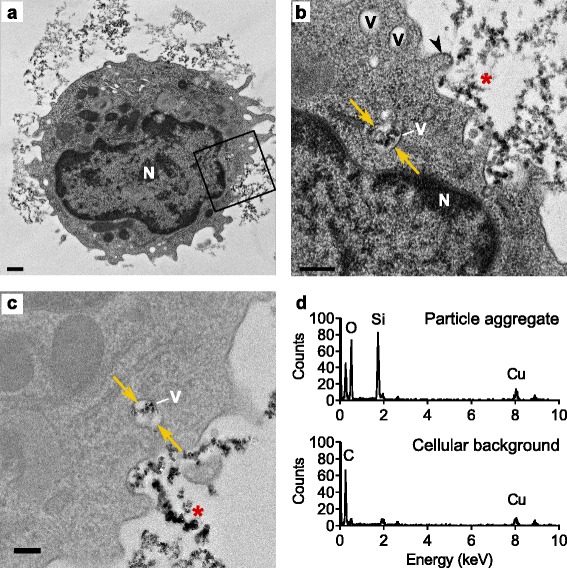
Internalization of nanomaterials by steady-state DCs. Immature DCs were incubated for 2 h at 37 °C with 250 μg ml^−1^ SAS particles (13-nm primary diameter) and analyzed by TEM. **a** Representative steady-state DC showing emerging dendrites interacting with particles. N, nucleus; bar, 0.5 μm. Contrasted with uranyl acetate/lead citrate for 15 min; the *rectangle* indicates the area selected for higher magnification. **b** Magnified region of the DC near its cell surface to highlight membrane protrusions (*arrowhead*) in the process of engulfing SAS particles (*asterisk*). The two *arrows* indicate internalized particles within a vacuole (V). Scale bar, 0.2 μm. **c** Magnified region of a DC that visualizes the process by which SAS particles (*asterisk*) are engulfed into intracellular vacuoles. A small SAS aggregate is enclosed in a vacuole. Scale bar, 0.2 μm; contrasted with uranyl acetate/lead citrate for 1 min to improve particle visibility. **d** Analysis of a representative intracellular SAS particle aggregate and cytoplasmic background by energy-dispersive X-ray spectroscopy (EDX). Internalized FePO_4_ and TiO_2_ particles are shown in Additional file 1: Figure S5

The uptake of food-grade SAS particles by immature DCs was further investigated by transmission electron microscopy (TEM) after high-pressure cryo-fixation to immobilize biological processes and visualize cellular components, including membranes, at nanometer resolution (Fig. 2a). This technique allows to confirm that the interaction of DCs with particles leads to true internalization as compared to adsorption on the cell surface. This electron microscopy approach showed that the irregularly shaped SAS particles form extracellular and intracellular aggregates. The electron micrographs also revealed multiple protruding dendrites on the cell surface (Fig. 2b) indicative of actin-dependent uptake mechanisms [36, 37]. By closure of these membrane protrusions, the SAS particle aggregates become trapped in cytoplasmic membrane-bound vesicles that have the appearance of endosomes (Fig. 2c). Analysis of membrane-enclosed particle aggregates by energy-dispersive X-ray spectroscopy (EDX) on a scanning TEM instrument confirmed their expected elemental composition, i.e. mainly silicon and oxygen (Fig. 2d and Additional file 1: Figure S4). As a control, analysis of the cellular background, not containing any SAS particles, revealed signals of carbon (a normal biological constituent and part of the embedding resin) and copper (from the TEM specimen support grid). Analogous images showed that immature DCs also internalize FePO_4_ and TiO_2_ nanoparticles and, as observed for SAS particles, localize them to membrane-bound vesicles with the appearance of endosomes (Additional file 1: Figure S5).

### Food-grade SAS particles cause induction of IL-1β in steady-state DCs

**Fig. 3:**
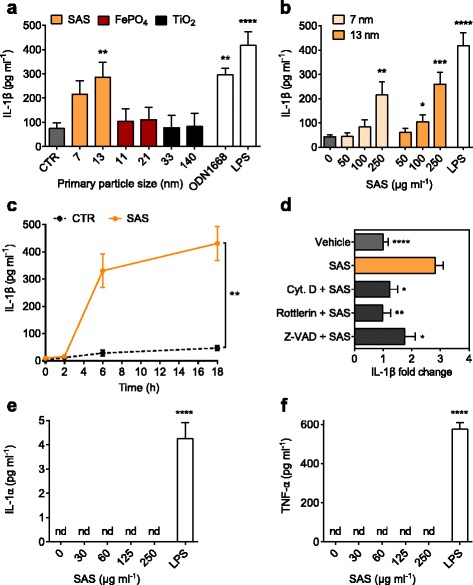
IL-1β secretion induced by food-grade SAS particles depends on uptake by macropinocytosis. Immature DCs were incubated (18 h, 37 °C) with particles to test for IL-1β secretion. Asterisks denote significant differences between SAS treatments and controls (*p < 0.05, **p < 0.01, ***p < 0.001, ****p < 0.0001). **a** DCs were exposed to particles (250 μg ml^−1^) and supernatants analyzed for IL-1β. Control reactions contained medium (CTR), 6 μg ml^−1^ ODN1668 (mimicking microbial DNA) or 1 μg ml^−1^ lipopolysaccharide (LPS). One-way ANOVA with Dunnet’s correction; *n* = 3–9; error bars, s.e.m. **b** Dose dependence of IL-1β secretion stimulated by SAS particles. Unpaired two-tailed t-test (*n* = 3–12). **c** Time dependence of IL-1β secretion stimulated by SAS particles (250 μg ml^−1^). Unpaired two-tailed t-test (*n* = 4). **d** Flt3L-generated immature DCs were incubated for 18 h at 37 °C with 13-nm SAS (250 μg ml^−1^) alone or in the presence of cytochalasin D (1.5 μg ml^−1^), rottlerin (1.5 μg ml^−1^) or Z-VAD (10 μg ml^−1^). Results represent fold changes relative to vehicle controls (one-way ANOVA with Dunnet’s correction, *n* = 3–12). **e** Release of IL-1α into the cell culture supernatant stimulated by LPS (1 μg ml^−1^) but not by 13-nm SAS particles. Asterisks denote significant differences between the LPS treatment and controls containing only culture medium. Cytokine levels below detection limit (4 pg ml^−1^) are indicated as not detectable (nd). One-way ANOVA with Dunnet’s correction; *n* = 4; error bars, s.e.m. **f** Release of TNF-α into the cell culture supernatant stimulated by LPS (1 μg ml^−1^) but not by 13-nm SAS particles (nd, cytokine levels below the detection limit of 8 pg ml^−1^. Asterisks denote significant differences between the LPS treatment and controls containing only culture medium. One-way ANOVA with Dunnet’s correction; *n* = 4; error bars, s.e.m.

To assess functional consequences of this nanomaterial uptake into immature DCs, we determined cell viability and secreted cytokine levels as biomarkers of inflammatory reactions. Propidium iodide staining demonstrated that immature DCs retained cell membrane integrity when incubated with SAS particles at concentrations up to 250 μg ml^−1^ (Additional file 1: Figure S6). However, SAS particles are able to initiate secretion of mature IL-1β from immature DCs even without any pre-stimulation, usually referred to as “priming”, with an inflammatory trigger (Fig. 3a). After 18-h incubations, we measured a low background IL-1β release (< 100 pg ml^−1^) in control reactions containing immature DCs and culture medium. The addition of 13-nm SAS particles was sufficient to increase this IL-1β production to levels of nearly 300 pg ml^−1^ or higher. All subsequent assays were performed with these more reactive 13-nm SAS particles.

Dose dependence experiments, again without DC priming, revealed a significantly increased IL-1β secretion into the cell culture medium at a SAS concentration of 100 μg ml^−1^ (Fig. 3b), corresponding to a surface-related nanomaterial density of 50 μg cm^−2^. In time course experiments, again without priming, significantly increased IL-1β secretion could be detected already following 6 h after the addition of SAS particles (Fig. 3c). Using small-molecule inhibitors, we next tested whether the observed intracellular particle uptake (Figs. 1 and 2) is necessary to induce cytokine release. IL-1β secretion was reduced to baseline levels upon co-treatment of DCs with SAS particles and cytochalasin D, which is a broad inhibitor of actin-dependent processes [12], or rottlerin, a selective inhibitor of macropinocytosis (Fig. 3d) [37]. These inhibitor effects support the conclusion that the IL-1β response to SAS particles requires prior endocytic uptake by actin-mediated mechanisms, primarily macropinocytosis. In contrast, despite their cellular uptake (Additional file 1: Figure S5), FePO_4_ and TiO_2_ nanoparticles elicit no IL-1β secretion (Fig. 3a). Thus, the observed initiation of cytokine release is a response that is specific to certain particles like food-grade SAS materials. In contrast to previous studies [26, 27], the extracellular secretion of IL-1β in response to SAS particles took place without any detectable release of the cytokines IL-1α (Fig. 3e) and TNF-α (Fig. 3f). Since contamination of the SAS batches with endotoxin was excluded by a sensitive *Limulus* amoebocyte lysate test (Table 1), it is concluded that food-grade SAS particles are sufficient to activate immature DCs and induce both the synthesis and release of a potent inflammatory cytokine even without preceding priming.

### Mechanism of IL-1β induction by SAS particles

**Fig. 4:**
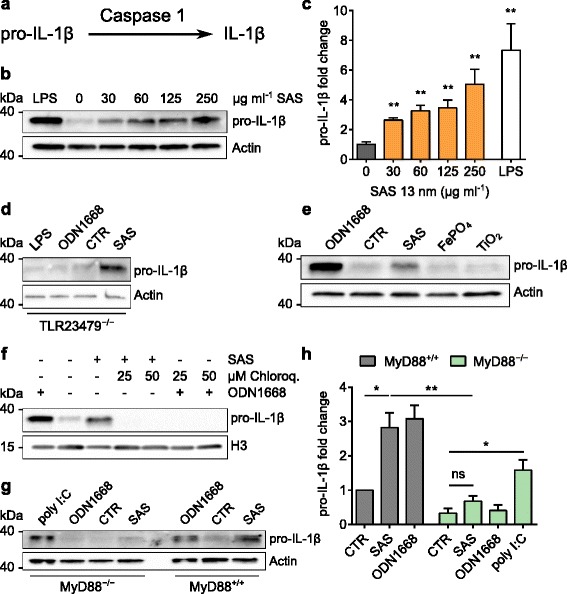
Induction of pro-IL-1β by food-grade SAS particles depends on MyD88. Immature DCs were incubated (18 h, 37 °C) with particles to test for IL-1β induction. Asterisks denote significant differences between SAS treatments and controls (*p < 0.05, **p < 0.01, ***p < 0.001, ****p < 0.0001). **a** Schematic illustrating the mechanism of IL-1β production. **b** DCs were incubated with LPS (250 ng ml^−1^) or 13-nm SAS and analyzed for pro-IL-1β (31 kDa) and actin (42 kDa) by immunoblotting. **c** Quantification of pro-IL-1β induction by SAS particles (unpaired two-tailed *t*-test, *n* = 5, error bars, s.e.m.). **d** Incubation of TLR2/3/4/7/9^−/−^ DCs with LPS (250 ng ml^−1^), ODN1668 (600 ng ml^−1^), medium (CTR) or SAS particles (125 μg ml^−1^). **e** Incubation of wild type DCs with ODN1668 (600 ng ml^−1^), medium or the indicated particles (125 μg ml^−1^). **f** Effect of endosomal TLR inhibition. DCs were incubated with 13-nm SAS (125 μg ml^−1^) alone or in the presence of chloroquine and analyzed for pro-IL-1β and histone H3 (17 kDa) by immunoblotting. ODN1668 (600 ng ml^−1^) served as positive control. **g** MyD88^−/−^ or wild type (MyD88^+/+^) DCs were incubated with poly I:C (5 μg ml^−1^), ODN1668 (600 ng ml^−1^), medium (CTR) or SAS particles (125 μg ml^−1^) and analyzed for pro-IL-1β (31 kDa) and actin (42 kDa) by immunoblotting. Split bands in some control lanes are an electrophoretic artifact not interfering with quantifications. **h** Pro-IL-1β induction by SAS particles in MyD88^−/−^ or wild type (MyD88^+/+^) DCs (unpaired two-tailed *t*-test, *n* = 3, error bars, s.e.m.)

Because of its potent inflammatory action, IL-1β biogenesis is tightly regulated. First, an inactive pro-IL-1β precursor is synthesized, which is then cleaved by the intracellular inflammasome complex (involving NLRP3 and caspase 1) to yield biologically active IL-1β (Fig. 4a) [23–25]. In line with this well-known process, the release of IL-1β upon stimulation with SAS particles was reduced partially by co-treatment of DCs with the caspase inhibitor Z-VAD (Fig. 3d), thus confirming that pro-IL-1β is caspase-cleaved to yield mature IL-1β [9, 21].

Next, we analyzed whole cell lysates for the presence of pro-IL-1β to understand whether this protein precursor occurs constitutively in immature DCs or is newly induced following SAS particle uptake. Immunoblot analyses using monoclonal antibodies against mouse pro-IL-1β showed that, in their immature state, steady-state DCs contain low levels of pro-IL-1β migrating in polyacrylamide gels as a band with an expected size of 31 kDa. In incubations of 18 h, however, SAS particles were able like lipopolysaccharide (LPS), a well-known inducer of pro-IL-1β used as positive control, to stimulate the de novo production of pro-IL-1β (Fig. 4b). Dose dependence experiments revealed a significantly increased pro-IL-1β level at a SAS concentration of 30 μg ml^−1^ (Fig. 4c), equivalent to a surface-related nanomaterial density of 15 μg cm^−2^. Pro-IL-1β was also induced upon a SAS treatment of immature DCs lacking Toll-like receptor 4 (TLR4; Additional file 1: Figure S7). As this particular pattern recognition receptor mediates DC activation by endotoxin [5], the retained pro-IL-1β induction observed in its absence confirms that the response is not due to endotoxin contamination. Further experiments showed that pro-IL-1β was equally induced upon SAS treatment of immature DCs lacking simultaneously TLR2, TLR3, TLR4, TLR7 and TLR9 (Fig. 4d). As these pattern recognition receptors mediate DC activation by pathogen constituents like endotoxin (TLR2 and TLR4) or nucleic acids (TLR3, TLR7 and TLR9) [5], the retained pro-IL-1β induction observed in their absence confirms that this response is not due to microbial contamination. In remarkable contrast but consistent with the results observed from the IL-1β secretion experiments (Fig. 3a), pro-IL-1β is not induced by FePO_4_ and TiO_2_ nanoparticles (Fig. 4e).

Considering that the particles are detected in vesicles with the appearance of endosomes (Fig. 2), the mechanism of pro-IL-1β induction by SAS was further delineated using endosomal acidification inhibitors. Pro-IL-1β induction by SAS particles was reduced to baseline levels upon co-treatment with chloroquine (Fig. 4f) or bafilomycin A1 (Additional file 1: Figure S8), which both inhibit the endosomal acidification process that is essential for TLR activation by pathogen-associated molecular patterns [38]. Stimulation of the nuclear transcription factor NF-κB in response to endosomal TLR activation, except for TLR3, further depends on the MyD88 adapter [5]. Therefore, we next tested the response of immature DCs lacking MyD88 (MyD88^−/−^) in comparison to wild type (MyD88^+/+^) controls. These experiments demonstrated that DCs lose the ability to induce pro-IL-1β in response to SAS exposure in the absence of MyD88 (Fig. 4g). To validate this finding, we needed to confirm that, like wild-type counterparts, MyD88-deficient DCs retained the ability to express IL-1β in response to an appropriate stimulus. This proof was provided by incubating these MyD88^−/−^ DCs with polyinosinic-polycytidylic acid (poly I:C), a synthetic analog of double-stranded RNA that activates TLR3, whose downstream signaling is independent of MyD88 [5]. A side-by-side quantification demonstrated that the induction of pro-IL-1β upon SAS exposure is totally missing in MyD88^−/−^ DCs (Fig. 4h). In combination, these experiments link for the first time an endosomal pattern recognition process to SAS particles.

### Display of maturation markers

**Fig. 5:**
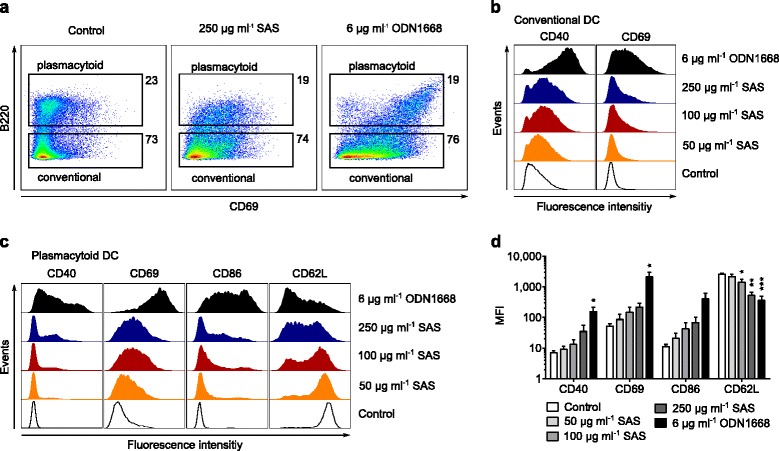
Maturation markers on steady-state DCs. Immature DCs were incubated for 18 h at 37 °C with the indicated stimulus and their surface markers were analyzed by flow cytometry. **a** Increased CD69 on the surface of DCs exposed to SAS particles (13-nm primary diameter) or oligonucleotide ODN1668. Plasmacytoid and conventional DCs are the two major subsets differing in B220 expression (numbers denote percentages of events in each gate). **b** Representative histograms showing dose-dependent changes of CD69 and CD40 on conventional DCs exposed to the indicated stimuli. Control, unstimulated DCs in culture medium. **c** Representative histograms showing dose-dependent changes in the display by CD69, CD40, CD86 and CD62L on plasmacytoid DCs. **d** Quantification of maturation markers by median fluorescence intensity (MFI) on plasmacytoid DCs exposed to SAS particles (13-nm primary size). Statistical significance (*p < 0.05, **p < 0.01, ***p < 0.001) was determined by one-way ANOVA with Dunnet’s correction, *n* = 3 experiments with independent bone marrow isolates; error bars, s.e.m

Immature DCs are distinguishable from those emerging during inflammation by their molecular phenotype [39]. For example, immature DCs are characterized by low surface expression of CD69, but the presentation of this maturation marker is increased upon incubation with SAS particles. CD69 up regulation occurs with both major subsets of steady-state DCs, i.e., with conventional and plasmacytoid DCs (Fig. 5a). Conventional DCs are further characterized by low CD40, which is increased upon exposure to SAS particles (Fig. 5b). Plasmacytoid DCs are further characterized by low CD40/CD86 and high CD62L, but incubation with SAS particles increased CD40 and CD86 on their surface, whereas CD62L levels were reduced (Fig. 5c and d). Thus, in response to SAS particles, immature DCs not only induce the synthesis of pro-IL-1β but also undergo a dose-dependent maturation program involving shifts in multiple surface markers exemplified by CD69, CD40, CD86 and CD62L. This finding confirms the ability of SAS particles to activate immature DCs.

## Discussion

This study is fundamentally different from earlier reports describing the interaction of DCs with nanomaterials in four main aspects. First, the ability of nanomaterials to trigger IL-1β secretion from DCs was previously demonstrated after pro-inflammatory priming of these cells to induce expression of the pro-IL-1β precursor. Consequently, these previous reports demonstrated that certain nanomaterials activate the inflammasome, which is an enzymatic complex that cleaves preformed pro-IL-1β to liberate active IL-1β [9, 12]. In contrast, we found that completely immature (unprimed) DCs respond to food-grade SAS particles by a de novo induction of the pro-IL-1β precursor. This response was absent when unprimed DCs were exposed to FePO_4_ and TiO_2_ nanoparticles. Second, we show that the newly discovered pro-IL-1β induction in response to food-grade SAS particles is suppressed by pharmacologic inhibitors of endosomal TLR activation. Third, our study reveals that pro-IL-1β induction in response to food-grade SAS particles is dependent on MyD88, an adapter protein essential for signaling downstream of members of the TLR family. So far, a role of MyD88 had been described for the inflammatory response to crystalline silica [40] but not to amorphous silica. Fourth, earlier reports were limited to inflammatory DCs derived from blood or bone marrow cultures stimulated with granulocyte-macrophage colony-stimulating factor (GM-CSF) [9, 12]. However, GM-CSF drives the differentiation of monocytes towards a special DC subset that sustains inflammation during exceptionally high demand like sepsis [1, 4, 41]. These inflammatory DCs differ from the Flt3L-derived immature DCs used in our study, whose function is to maintain tissue homeostasis by tuning immunologic responses [1, 2, 32]. It is known that particle sedimentation rates influence the amount of material that comes in contact with cells. Existing simulation models [42, 43] are limited to the exposure of cell monolayers and not suited to estimate effective doses in cell suspensions. Therefore, we experimentally compared the cellular dose by exploiting shifts of the SSC parameter in flow cytometry analyses (Fig. 1). This comparison shows that the reactions elicited by SAS particles, but not by FePO_4_ and TiO_2_ particles, cannot be explained by dosimetric considerations, since a slightly higher concentration of the SAS material was required to reach the same cell exposure as that reached with FePO_4_ and TiO_2_ particles.

Protective immunity is supported by IL-1β but, if not controlled, this highly inflammatory cytokine may contribute to autoinflammatory and autoimmune diseases [25]. Normally, IL-1β production is regulated by two independent signals. A first stimulus, such as LPS, microbial nucleic acids, IL-1α or TNF-α, prompts the synthesis of inactive pro-IL-1β by transcriptional induction [26, 27]. A second stimulus leads to the assembly of a large multiprotein complex termed inflammasome that, upon sensing exogenous pathogen-associated molecular patterns or endogenous stress-associated danger signals, cleaves pro-IL-1β to release mature IL-1β [9, 12, 21, 23, 24]. In a model of acute pulmonary inflammation, Rabolli et al. describe a sequential mechanism in which lung-injected silica is taken up by macrophages, thus leading to cytotoxicity [26]. The following release of IL-1α and TNF-α from necrotic macrophages stimulates the expression of pro-IL-1β, which is then cleaved upon silica-dependent inflammasome activation for the secretion of active IL-1β [26]. In contrast to this sequential scenario, our present analysis with immature DCs (reflecting steady-state DCs in tissues) reveals a one-hit mechanism whereby food grade SAS particles induce pro-IL-1β expression in the absence of cytotoxic reactions and IL-1α or TNF-α bursts.

This direct pro-IL-1β induction depends on MyD88, which is the central adapter that conveys endosomal TLR-signaling to induce pro-inflammatory cytokines via NFκB activation [6, 7], but pro-IL-1β induction is not dependent on the pattern recognition receptors TLR2, TLR3, TLR4, TLR7 and TLR9. The acidification of endosomal contents is an essential process for TLR activation by pathogens [38]. Thus, the finding that endosomal acidification inhibitors like chloroquine or bafilomycin A completely abrogate pro-IL-1β induction by food-grade SAS particles indicates that an endosomal pattern recognition receptor, likely another TLR, must constitute their molecular sensor. In any case, this proven dependence on endosomal acidification and MyD88 signaling argues against other mechanisms for example associated with cytotoxicity or increased K^+^ efflux through the cell membrane [44, 45].

**Fig. 6:**
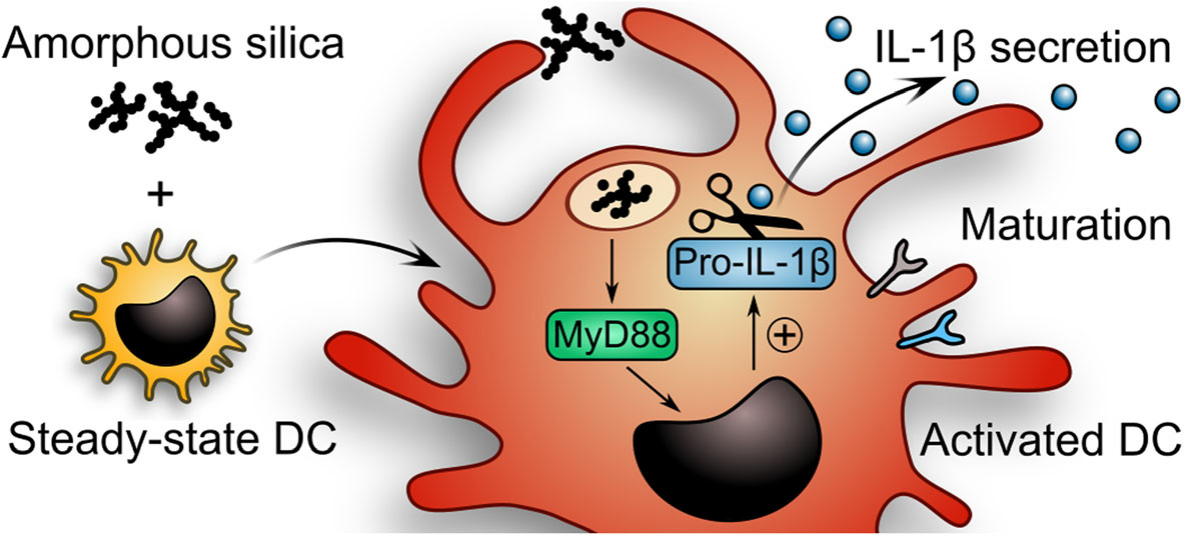
Scheme summarizing the single-hit mechanism of IL-1β secretion by steady-state DCs exposed to food-grade SAS particles. In immature DCs, pro-IL-1β is not expressed, but its induction takes place upon uptake of SAS particles into endosomes and MyD88-dependent TLR signaling. Cleavage of the newly expressed pro-IL-1β precursor by the inflammasome-associated caspase leads to IL-1β secretion. In addition, the immature DCs undergo maturation involving shifts in multiple surface markers. IL-1β secretion in response to SAS particles is suppressed by cytochalasin or rottlerin (inhibitors of actin-dependent macropinocytosis), by chloroquine or bafilomycin A (inhibitors of endosomal TLR activation), by genetic ablation of MyD88 (the central adapter of TLR signaling) and by Z-VAD (an inhibitor of the inflammasome-associated caspase)

As summarized in Fig. 6, our findings indicate that steady-state DCs take up nanostructured SAS particles by actin-dependent macropinocytosis, inhibited by cytochalasin and rottlerin (Fig. 3d), and localize the particles to endosomal vesicles (Fig. 2). Upon endosomal acidification, inhibited by chloroquine or bafilomycin A (Fig. 4g), the unique nanostructured surface pattern of SAS particle aggregates is recognized by TLRs on endosomal membranes. This unexpected TLR engagement is highlighted by the finding that the SAS particle-induced pro-IL-1β synthesis is suppressed upon deletion of MyD88 (Fig. 4g and h), which for most TLRs constitutes the obligate mediator of downstream signaling. In steady-state DCs, the tested food-grade SAS particles mimic both the signal for inducing pro-IL-1β expression (Fig. 4) and the signal for activating pro-IL-1β cleavage to release IL-1β (Fig. 3). With respect to the concentration used for in vitro experiments, it is important to consider the effective local dose of particles that comes in contact with target cells in the organism [46]. We used a realistic range because, even if only a small fraction of daily ingested SAS materials accumulates over time in lymphoid tissues [28, 29, 31], persisting particles may reach locally in the intestinal lymphoid tissue the 15 μg cm^−2^ density that in our experiments is sufficient for a marked IL-1β induction.

## Conclusion

Nanostructured SAS particles initiate the endosomal MyD88 pathogen pattern recognition and signaling pathway in steady-state DCs. The resulting de novo DC activation and pro-IL-1β induction, without the need for any concomitant cytotoxic or inflammatory trigger, suggests that SAS particles should be used in food more cautiously than current practice. Based on studies in rodents not assessing immunological endpoints, a lifelong dietary intake of 1.5 g SAS daily (Additional file 1: Figure S9) per adult is currently considered safe (reviewed in [47]). It has been suggested that a disruption of the gatekeeping function of DCs may trigger long-term inflammatory responses that contribute to the increased incidence of inflammatory bowel disease or other chronic intestinal disorders [11, 28]. It is still necessary to carefully ascertain, in animal studies focused on reactions of the gut-associated lymphoid tissue, whether the newly discovered DC activation and pro-IL-1β induction by food-grade SAS particles occurs in vivo and causes adverse effects. It is for example possible that a particle-induced IL-1β release is counteracted in the context of the intestinal mucosa by the presence of IL-1β receptor antagonist. In any case, in view of the unexpected one-hit mechanism of cytokine production elicited by food-grade SAS particles in steady-state DCs, we advocate a prudent use of these and related nanomaterials to avoid excessive human exposure from food.

## Methods

### Particles and Characterization

Food-grade SAS particles, produced by flame hydrolysis according to the Aerosil method [48], were obtained from Evonik (formerly Degussa). Their mean primary particle sizes are 7 and 13 nm based on measured BET surface areas of 326 m^2^ g^−1^ and 175 m^2^ g^−1^, respectively. These same SAS particles were also characterized thoroughly by others [15, 16]. TiO_2_ anatase with an average particle size of 33 nm (based on a measured BET surface area of 47 m^2^ g^−1^) were from Sigma-Aldrich. Food-grade TiO_2_ anatase with an average particle size of 140 nm (based on a measured BET surface area of 11 m^2^ g^−1^) was from Sachtleben. FePO_4_ nanoparticles with an average particle size of 11 and 21 nm (based on measured BET surface areas of 188 m^2^ g^−1^ and 98 m^2^ g^−1^, respectively) were produced by flame spray pyrolysis according to a published method [19] with minor adaptations to obtain the desired particle sizes. Iron nitrate nonahydrate (purity ≥97.0%, Riedel-de-Haën/Sigma-Aldrich) and tributyl phosphate (purity 97%; Sigma-Aldrich) were dissolved in a 1:1 (vol/vol) mixture of ethanol (abs. Denat. 2% 2-butanone, Alcosuisse) and 2-ethylhexanoic acid (purity ≥99%; Sigma-Aldrich) at a total metal concentration of 0.5 mol l^−1^ or 0.4 mol l^−1^ for 21-nm FePO_4_ and 11-nm FePO_4_, respectively. This precursor solution was fed at 3 or 7 ml min^−1^ into the FSP spray nozzles by a syringe pump (Lambda, VIT-FIT) and atomized with 5 or 7 l min^−1^ oxygen (pressure drop 1.5 bar). The spray was ignited by a methane/oxygen (2.5 l min^−1^ each) ring-shaped flame. Additionally, 5 l min^−1^ (sheath) O_2_ was supplied. All gas flow rates were regulated by mass flow controllers (Bronkhorst, EL-FLOW). Using a vacuum pump (Busch, Mink MM1202 AV), particles were collected on water-cooled teflon membrane-filters (BHA Technologies AG) placed at least 65 cm above the burner. Amino-modified PS particles with a primary particle diameter of 50 nm were obtained from Bangs Laboratories. The detailed characterization of all nanomaterials is summarized in Table 1.

For visualization of particle shape by TEM, the nanomaterial were dispersed in H_2_O, deposited on a parlodion foil supported on a copper grid and analyzed on a CM12 microscope (FEI, operated at an acceleration voltage of 100 kV). For endotoxin analyses, particles were suspended in endotoxin-free water (Charles River) by mixing for 30 s on a vortex followed by sonication for 10 min in a Sonorex Digitec waterbath (Bandelin Electronic) at 35 kHz and 80 W. Then particle suspension was assayed using the Endosafe-PTS system (Charles River) equipped with highest sensitivity cartridges with a limit of detection of 0.005 endotoxin units (EU) ml^–1^. This validated test controls for interferences with colorimetric measurements using two internal endotoxin spikes according to guidelines of the Food and Drug Administration. In particular, the acceptance range for spike recovery of 50–200% excludes that the tested particles interfere with the assay by assuring that there is no signal inhibition or enhancement due to particles [49]. For exposure of cells, particles were suspended in complete cell culture medium [RPMI-1640 GlutaMAX, 10% (vol/vol) heat-inactivated fetal calf serum (from PAA), 100 U ml^–1^ penicillin, 100 μg ml^–1^ streptomycin and 50 μM β-mercaptoethanol (all from Invitrogen)] by mixing and sonication as described above. Fresh particle suspensions were prepared for every experiment and used within 15 min after sonication. SAS suspensions in complete medium remained stable for 24 h, as assessed by hydrodynamic size measurements.

### Stimulation of Immature DCs

Immature DCs were generated from mouse bone marrow [33]. Briefly, femurs and tibiae of C57BL/6 wild-type mice (or MyD88- and TLR-deficient mice in a C57BL/6 background) were flushed with culture medium and the released progenitor cells were filtered through a 70-μm cell strainer (BD Falcon), centrifuged, reconstituted in complete medium and incubated for 8 days in the presence of 200 ng ml^−1^ Flt3L (BioXcell). For each culture, the amount of live CD11c^+^ DCs, the proportion of plasmacytoid DCs (CD11c^+^B220^+^) and conventional DCs (CD11c^+^B220^−^CD11b^high^SIRPα^+^ and CD11c^+^ B220^−^CD11b^intermediate^SIRPα^−^) were verified by flow cytometry (Additional file 1: Fig. S1). Stimulation assays were conducted as previously published [50]. Immature DCs, which grow as cell suspensions, were transferred to 96-well plates (3 × 10^5^ cells/well) containing complete cell culture medium (200 μl/well) and challenged with the indicated concentrations of particles suspended in medium for the time indicated. Realistic SAS particle dose concentrations (30 to 250 μg ml^−1^) were derived from a recent publication, where silica concentrations of up to 300 μg g^−1^ tissue in the spleen of rodents after repeated oral administration of SAS particles were reported [31].

Control incubations were carried out with ultra-pure LPS (from *Escherichia coli* 0111:B4*,* Sigma-Aldrich), polyinosinic-polycytidylic acid (poly I:C, InvivoGen) or unmethylated deoxyribonucleic acid (DNA) oligonucleotides containing a CpG motif (ODN1668, TIB Molbiol). The inhibitors bafilomycin A1 (InvivoGen), cytochalasin D (Enzo Life Sciences), rottlerin (Sigma-Aldrich) and Z-VAD-FMK (Bachem) were dissolved in dimethyl sulfoxide and added as indicated. Chloroquine diphosphate salt (Sigma-Aldrich) was added as indicated.

### DC Phenotype and Maturation

DCs were stained on ice with conjugated antibodies against CD11c (N418, PE-labeled), CD11b (M1/70, PE-Cy7-labeled), B220 (RA3-6B2, APC-eFluor 780-labeled), SIRPα (P84, APC-labeled), CD40 (1C10, PE-labeled), CD62L (MEL-14, APC-labeled), CD69 (H1.2F3, APC-labeled) and CD86 (GL1, PE-labeled) purchased from eBioscience. A FACSCanto II flow cytometry instrument (BD Biosciences) was employed to acquire 50,000 events. Dead cells were stained and excluded from analyses using propidium iodide (PI; Sigma-Aldrich). Single-color and fluorescence-minus-one (FMO) controls were prepared and analyzed along with multi-color samples. Flow cytometry data were analyzed with FlowJo (Version 10, TreeStar).

### Cryo-fixation and Electron Microscopy

Immature DCs were transferred to 12-well plates (3 × 10^6^ cells/well) containing cell culture medium (1 ml/well). After incubation with particles (250 μg ml^−1^), cells were prefixed with 0.25% (vol/vol) glutaraldehyde and immediately high-pressure frozen in capillary cellulose tubes using an EM HPM 100 device (Leica). Frozen cells were transferred into a substitution unit (EM-AFS2, Leica) precooled to −90 °C for substitution with acetone containing 5% water. The subsequent fixation was carried out with 1% (wt/vol) osmium tetraoxide, 0.25% (vol/vol) glutaraldehyde raising the temperature to 20 °C, then the cells were embedded in epon. Ultrathin (70 nm) sections were contrasted with uranyl acetate and lead citrate for 1 or 15 min [51] and examined in a transmission electron microscope (CM12, Philips) equipped with a CCD camera (Ultrascan 1000, Gatan) at an acceleration voltage of 100 kV. Elemental analysis of selected samples was conducted on a scanning transmission electron microscope (G2 Spirit, FEI Tecnai) equipped with a high angle annular dark field detector (HAADF) and an X-Max energy-dispersive X-ray spectroscopy (EDX) detection system for elemental analysis (Oxford). Gatan digital micrograph was used for image acquisition and Oxford INCA for EDX operation and control.

### Immunoassays

IL-1β concentrations in cell culture supernatants were detected using the IL-1β DuoSet kit (R&D Systems) following the manufacturer’s instructions. Absorbance was measured at 405-nm wavelength (reference wavelength 492 nm) with a SpectraMax Plus 384 microplate reader (Molecular Devices). Mouse IL-1α and TNF-α concentrations in cell culture supernatants were detected using the IL-1α and TNF-α Ready-SET-Go kits (eBioscience) following the manufacturer’s instructions. Absorbance was measured at 450-nm wavelength (reference wavelength 570 nm) with a Epoch 2 microplate reader (BioTek). For Western blotting, cells were washed three times with phosphate-buffered saline (PBS) and whole cell lysates were prepared using M-PER buffer (LifeTechnologies). Equal volumes of cell lysate were resolved on 4–20% (wt/vol) polyacrylamide gradient gels (TGX Stainfree) and the separated proteins were transferred to TurboBlot PVDF membranes (both from BioRad). For immunodetection, membranes were blocked with 10% (wt/vol) milk in Tris-buffered saline, containing 0.1% (vol/vol) Tween-20, for 1 h at room temperature and probed overnight with monoclonal antibodies against mouse IL-1β (Cell Signaling) and actin (Millipore). After incubation with matching secondary antibodies, chemiluminescence was detected using the ChemiDoc MP gel documentation instrument (BioRad).

### Statistical Analysis

Mean and standard error of the mean (s.e.m.) were calculated for all quantitative parameters using GraphPad Prism 6.0. Results were expressed as mean ± s.e.m. of multiple determinations with independent DC cultures. Comparisons were conducted by one-way ANOVA with Dunnet’s correction or unpaired two-tailed *t*-test, as indicated in the figure legends. A statistically significant difference was assumed for *p* < 0.05.

## Additional file

Additional file 1: Characterization of steady-state DCs (**Figure S1**). TEM analysis of SAS, FePO_4_ and TiO_2_ particles (**Figure S2**). Interaction of steady-state DCs with FePO_4_ and TiO_2_ particles (**Figure S3**). Scanning TEM analysis of SAS internalization by steady-state DCs (**Figure S4**). Internalization of FePO_4_ and TiO_2_ nanoparticles by steady-state DCs (**Figure S5**). Cell viability upon incubation with SAS particles (**Figure S6**). Induction of pro-IL-1β by SAS particles in TLR4^−/−^ DCs (**Figure S7**). Effect of bafilomycin A1 on pro-IL-1β induction by SAS particles (**Figure S8**). Safe upper limit of nano-structured SAS particles. (**Figure S9**). (DOCX 4238 kb)

## Abbreviations

BET: Brunauer-Emmett-Teller theory
CpG: DNA oligonucleotide with unmethylated cytosine-phosphatidyl-guanine motif
DC: dendritic cell
DNA: deoxyribonucleic acid
EDX: energy-dispersive X-ray spectroscopy
ELISA: enzyme-linked immuno sorbent assay
EU: endotoxin units
Flt3L: feline McDonough sarcoma-like tyrosine kinase 3 ligand
FMO: fluorescence-minus-one
FSC: front scatter
GM-CSF: granulocyte-macrophage colony-stimulating factor
HAADF: high angle annular dark filed detector
IL: interleukin
LOD: limit of detection
LPS: lipopolysaccharide
MyD88: myeloid differentiation primary response gene 88
NLR: NOD-like receptor
ODN: DNA oligonucleotide
PBS: phosphate-buffered saline
PS: polystyrene
SAS: synthetic amorphous silica
SSC: side scatter
TEM: transmission electron microscopy
TLR: Toll-like receptor
TNF-α: tumor necrosis factor-α
