# Supplementary material for: MyD88-dependent pro-interleukin-1β induction in dendritic cells exposed to food-grade synthetic amorphous silica
Source: Part Fibre Toxicol. 2017 Jun 23;14:21. doi: 10.1186/s12989-017-0202-8 (PMC5481969; doi:10.1186/s12989-017-0202-8)
Supplement: Additional file 1: — Characterization of steady-state DCs (Figure S1). TEM analysis of SAS, FePO4 and TiO2 particles (Figure S2). Interaction of steady-state DCs with FePO4 and TiO2 particles (Figure S3). Scanning TEM analysis of SAS internalization by steady-state DCs (Figure S4). Internalization of FePO4 and TiO2 nanoparticles by steady-state DCs (Figure S5). Cell viability upon incubation with SAS particles (Figure S6). Induction of pro-IL-1β by SAS particles in TLR4−/− DCs (Figure S7). Effect of bafilomycin A1 on pro-IL-1β induction by SAS particles (Figure S8). Safe upper limit of nano-structured SAS particles. (Figure S9). (DOCX 4238 kb) [file 12989_2017_202_MOESM1_ESM.docx]

**ADDITIONAL FILE 1**

**MyD88-dependent pro-interleukin-1β induction in dendritic cells exposed to food-grade synthetic amorphous silica**

*Hans Christian Winkler^1§^, Julian Kornprobst^1^, Peter Wick^2^, Lea Maria von Moos^3^, Ioannis Trantakis^3^, Elisabeth Maria Schraner^4^, Barbara Bathke^5^, Hubertus Hochrein^5^, Mark Suter^6^, Hanspeter Naegeli^1*^*

^1^Institute of Pharmacology and Toxicology, University of Zurich-Vetsuisse, Winterthurerstrasse 260, 8057 Zurich, Switzerland.

^2^Laboratory for Particles-Biology Interactions, Empa Swiss Laboratories for Materials and Technology, Lerchenfeldstrasse 5, 9014 St. Gallen, Switzerland.

^3^Department of Health Sciences and Technology, ETH Zurich, Schmelzbergstrasse 9, 8092 Zurich, Switzerland.

^4^Electron Microscopy, Institutes of Veterinary Anatomy and Virology, Winterthurerstrasse 260, 8057 Zurich, Switzerland.

^5^Department of Research, Bavarian Nordic GmbH, 82152 Martinsried, Germany.

^6^Immunology Division, Vetsuisse Faculty, University of Zurich, Winterthurerstrasse 204, 8057 Zürich, Switzerland.

^§^Present address: Institute of Food, Nutrition and Health, Laboratory of Human Nutrition, ETH Zurich, Schmelzbergstrasse 7, 8092 Zurich, Switzerland.

*Correspondence to: [naegelih@vetpharm.uzh.ch](mailto:naegelih@vetpharm.uzh.ch)


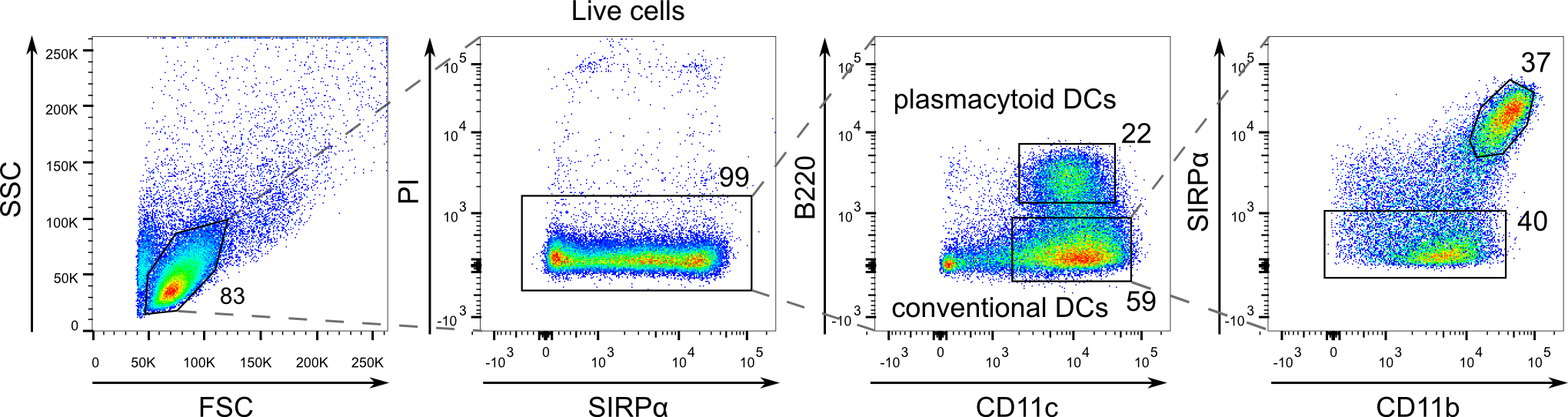


Figure S1. Characterization of steady-state DCs.

Immature DCs, generated from mouse bone marrow progenitors in the presence of Flt3L, were analyzed by flow cytometry. For each culture, the proportion of live CD11c^+^ DCs, the proportion of plasmacytoid DCs (CD11c^+^B220^+^) and the proportion of conventional DCs (CD11c^+^B220^–^CD11b^high^SIRPα^+^ and CD11c^+^ B220^–^CD11b^intermediate^SIRPα^-^) were verified using the respective antibodies. Numbers indicate the percentage of events in each gate. PI, propidium iodide.


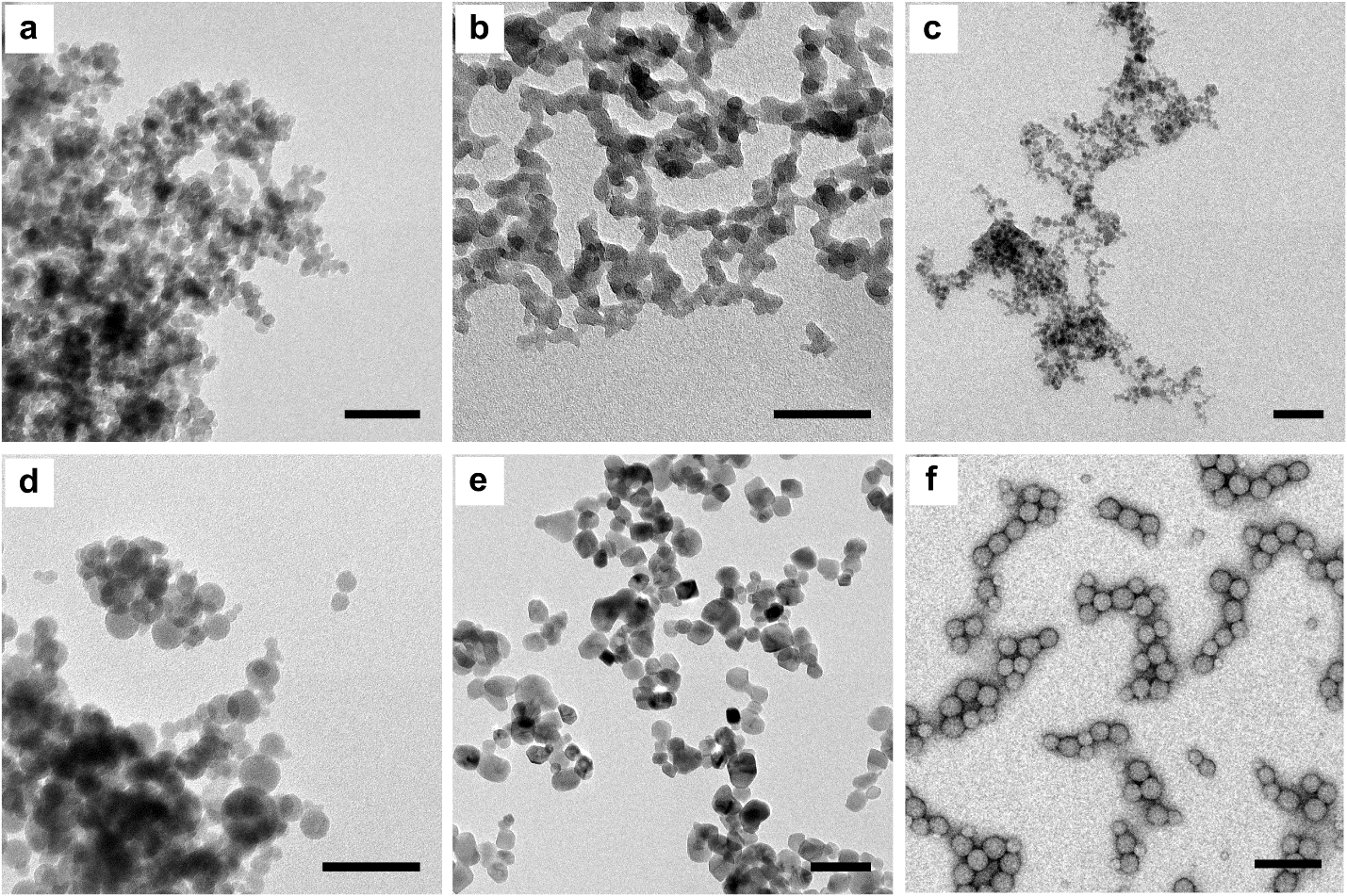


Figure S2. TEM analysis of SAS, FePO_4_ and TiO_2_ particles.

SAS, FePO_4_ and TiO_2_ particles were dissolved in H_2_O and analyzed by TEM. (**a**) SAS (7-nm primary particle diameter). Scale bar, 0.1 µm. (**b**) SAS (13-nm primary particle diameter). Scale bar, 0.1 µm. (**c**) FePO_4_ (11-nm primary particle diameter). Scale bar, 0.1 µm. (**d**) FePO_4_ (21-nm primary particle diameter). Scale bar, 0.1 µm. (**e**) TiO_2_ (33-nm primary particle diameter). Scale bar, 0.1 µm. (**f**) PS size standards with 60-nm primary particle diameter. Scale bar, 0.2 µm.
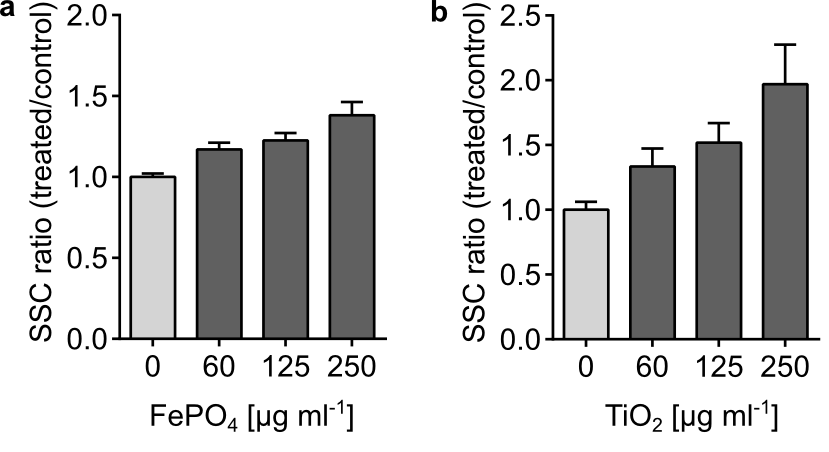


Figure S3. Interaction of steady-state DCs with FePO_4_ and TiO_2_ particles.

Flt3L-generated immature DCs were incubated for 1 h at 37°C with the indicated concentrations of FePO_4_ (11-nm diameter) or TiO_2_ particles (33-nm diameter) suspended in cell culture medium, and analyzed by flow cytometry. The side scatter (SSC) signal reflects an increment of intracellular structures due to particle uptake. (**a**) Ratios of median SSC values resulting from incubation of immature DCs with FePO_4_ relative to controls. (**b**) Ratios of median SSC values obtained from incubation of immature DCs with TiO_2_ particles relative to controls. Upon one-way ANOVA, SSC values resulting from the incubation of immature DCs with FePO_4_ and TiO_2_ particles were significantly higher than controls (*p*<0.05, n = 3 experiments with independent bone marrow isolates, error bars indicate s.e.m.).


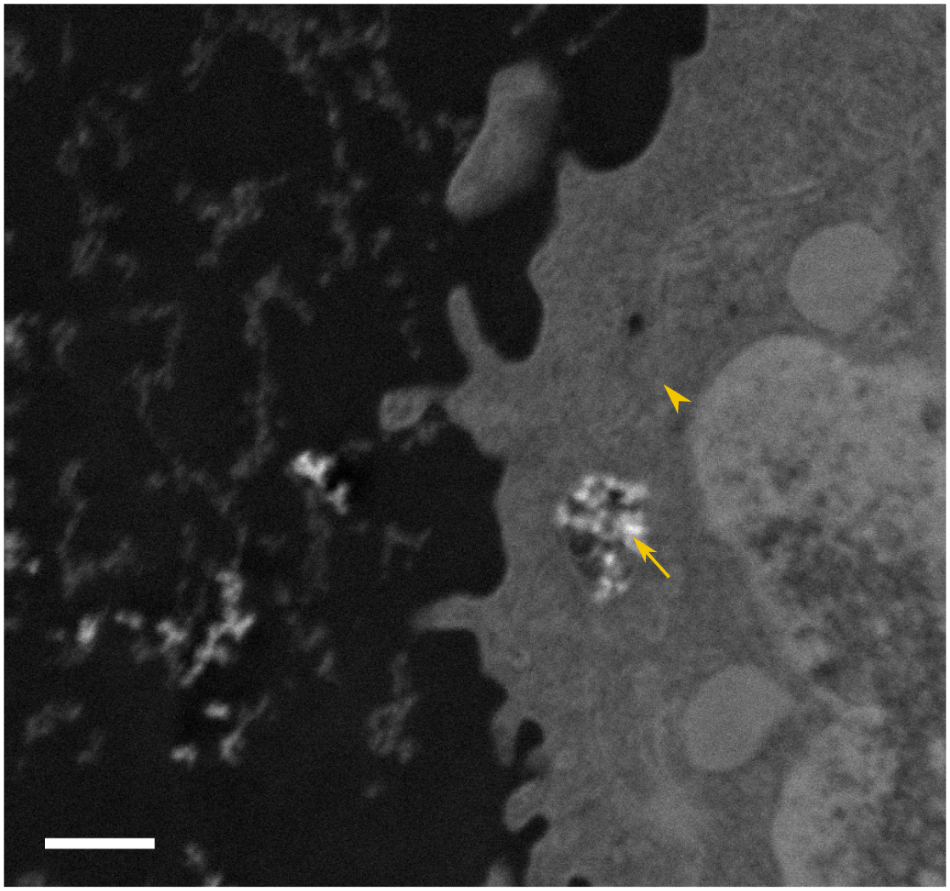


Figure S4. Scanning TEM analysis of SAS internalization by steady-state DCs.

Immature DCs were incubated for 2 h at 37°C with 250 µg ml^–1^ SAS particles (13-nm primary diameter) and analyzed by scanning TEM. The arrow and arrowhead indicate the points of interest, from where the spectra of intracellular SAS particle aggregates and the cytoplasmic background respectively were obtained by energy-dispersive X-ray spectroscopy (EDX). Scale bar, 0.5 µm. The corresponding spectra are shown in Figure 2d.


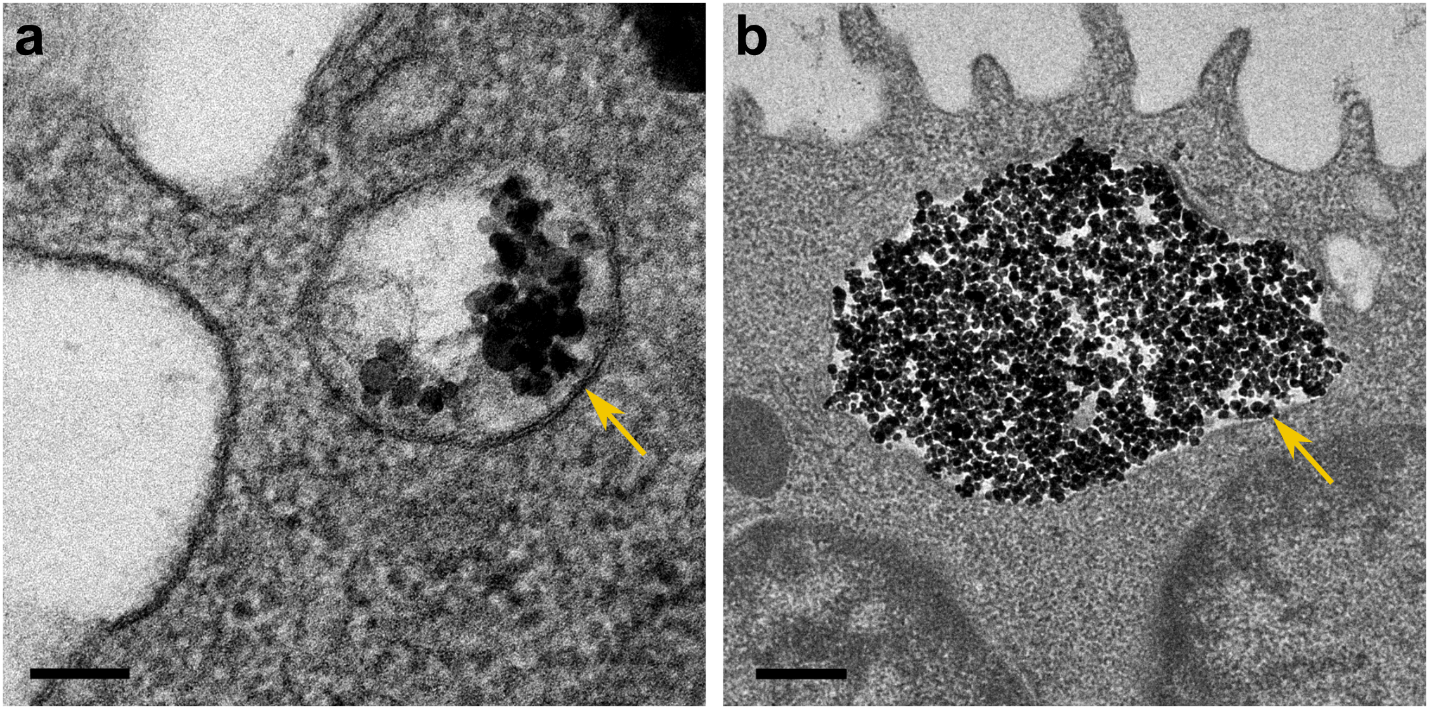


Figure S5. Internalization of FePO_4_ and TiO_2_ nanoparticles by steady-state DCs.

Flt3L-generated immature DCs were incubated for 2 h at 37°C with 250 µg ml^–1^ TiO_2_ or FePO_4_ particles and analyzed by transmission electron microscopy. (**a**) Typical steady-state DC interacting with FePO_4_ (11-nm primary particle diameter). The arrow indicates internalized FePO_4_ particles within a cytoplasmic membrane defining a vacuole. Scale bar, 0.1 µm. (**b**) Typical steady-state DC interacting with TiO_2_ (33-nm primary particle diameter). The arrow indicates internalized TiO_2_ particles within a cytoplasmic membrane defining a vacuole. Scale bar, 0.5 µm.


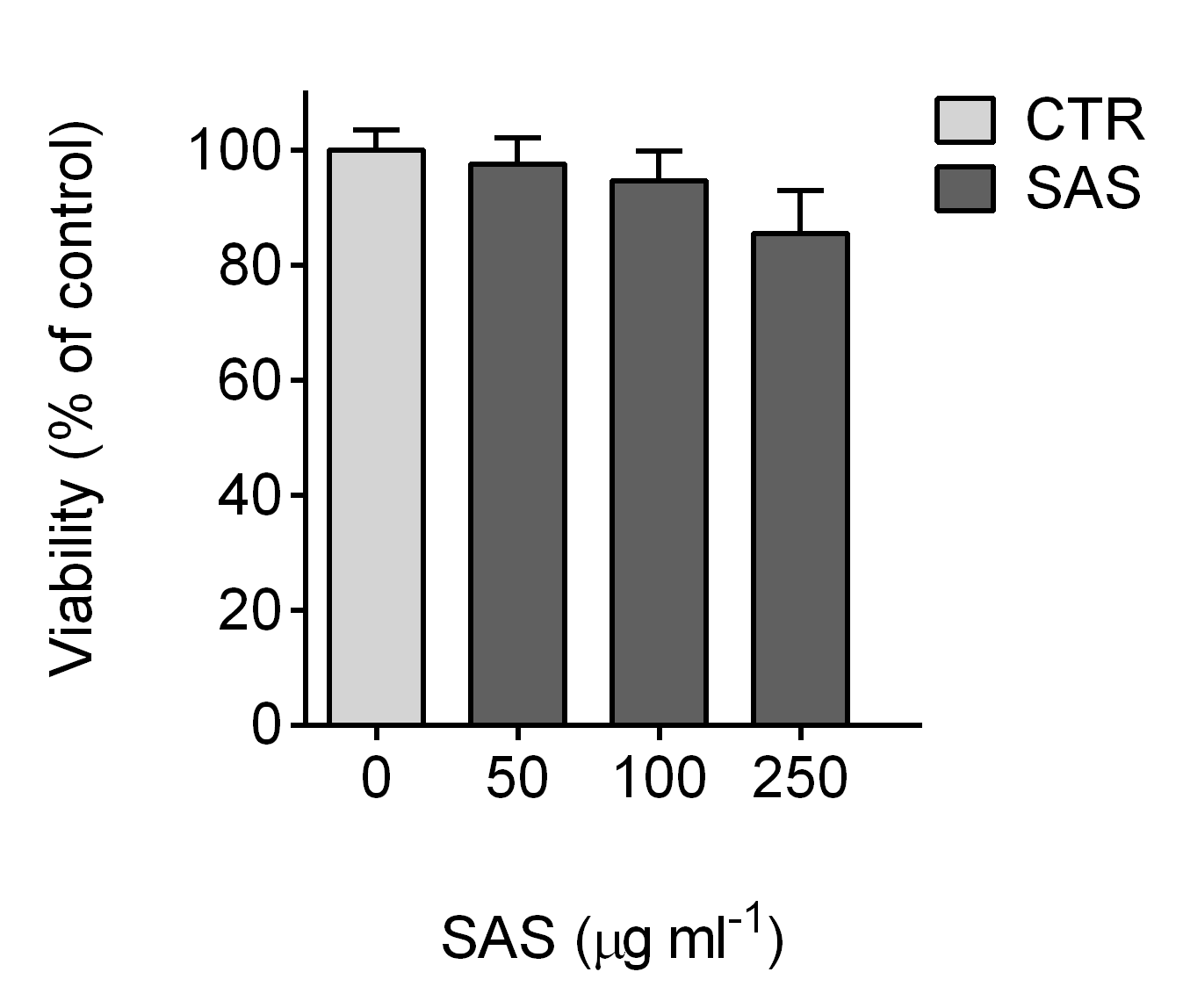


Figure S6. Cell viability upon incubation with SAS particles.

The effect of 13-nm SAS particles on cell viability was analyzed by flow cytometry after propidium iodide staining. Flt3L-generated immature DCs were incubated for 18 h at 37°C with 13-nm SAS particles at the indicated concentrations. Results represent the percentage of viable (propidium iodide-negative) cells relative to medium only control. Upon one-way ANOVA, the viability values resulting from the incubation of immature DCs with SAS particles were not significantly different from controls (one-way ANOVA with Dunnet's correction, n = 5, error bars indicate s.e.m.). Please note that a statistically significant pro-IL-1β induction is observed at a SAS dose of 30 µg ml^-1^.


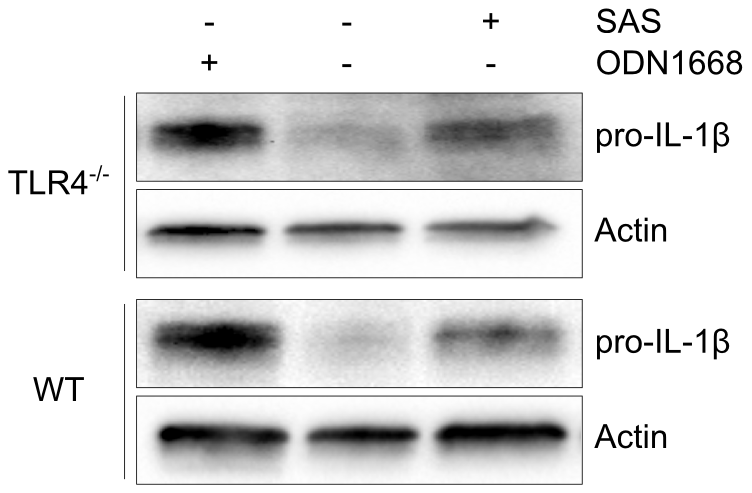


Figure S7. Induction of pro-IL-1β by SAS particles in TLR4^–/–^ DCs.

Flt3L-generated immature DCs from wildtype (WT) or TLR4^–/–^ mice were incubated for 18 h at 37°C with 13-nm SAS (125 µg ml^–1^) in cell culture medium, and analyzed by immunoblotting. Control reactions contained medium alone or 600 ng ml^–1^ ODN1668 (mimicking microbial DNA).


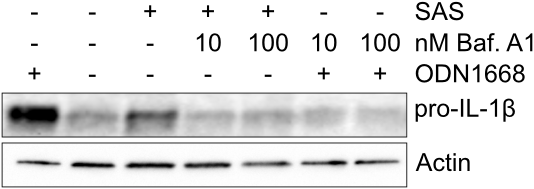


Figure S8. Effect of bafilomycin A1 on pro-IL-1β induction by SAS particles.

Flt3L-generated immature DCs from wildtype mice were incubated for 18 h at 37°C with 13-nm SAS (125 µg ml^–1^) alone or in the presence of bafilomycin A1 (10 or 100 nM), and analyzed by immunoblotting for pro-IL-1β. Control reactions contained medium alone or 600 ng ml^–1^ ODN1668 (mimicking microbial DNA).

**
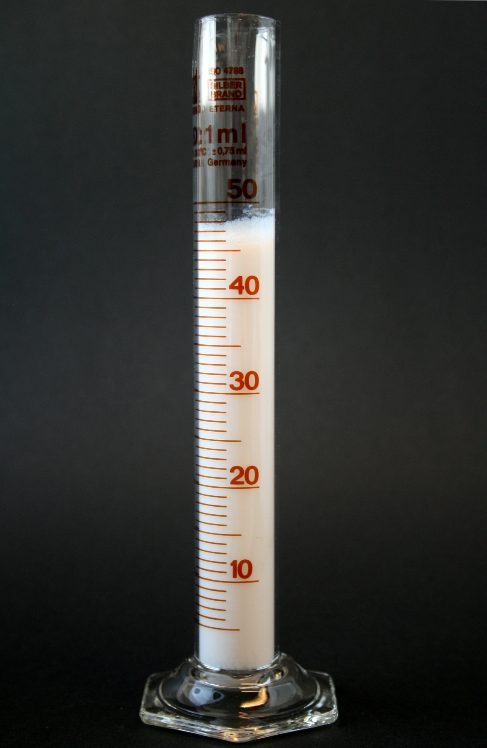
**

**Figure S9. Safe upper limit of nano-structured SAS particles.**

The 50-ml measuring cylinder contains 1.5 g of a white fluffy powder consisting of SAS particles produced by the Aerosil method. This amount of food-grade SAS material is currently considered safe for a 60-kg adult if consumed daily for a lifetime as food additive [1,2].

**Supplementary references:**

1. van Kesteren PCE, Cubadda F, Bouwmeester H, van Eijkeren JCH, Dekkers S, de Jong WH, et al. Novel insights into the risk assessment of the nanomaterial synthetic amorphous silica, additive E551, in food. Nanotoxicology. 2015;9:442–52.

2. EFSA 2009. European Food Safety Authority. Scientific opinion of the panel on food additives and nutrient sources added to food on calcium silicate, silicon dioxide and silicic acid gel added for nutritional purposes to food supplements following a request from the European Commission. EFSA J. 2009;1132:1–24.
